# Amyloid β influences the relationship between cortical thickness and vascular load

**DOI:** 10.1002/dad2.12022

**Published:** 2020-04-17

**Authors:** Thomas D. Parker, David M. Cash, Christopher A. Lane, Kirsty Lu, Ian B. Malone, Jennifer M. Nicholas, Sarah‐Naomi James, Ashvini Keshavan, Heidi Murray‐Smith, Andrew Wong, Sarah M. Buchanan, Sarah E. Keuss, Carole H. Sudre, David L. Thomas, Sebastian J. Crutch, Nick C. Fox, Marcus Richards, Jonathan M. Schott

**Affiliations:** ^1^ Department of Neurodegenerative Disease The Dementia Research Centre, UCL Queen Square Institute of Neurology London UK; ^2^ Department of Medical Statistics London School of Hygiene and Tropical Medicine London UK; ^3^ School of Biomedical Engineering and Imaging Sciences King's College London London UK; ^4^ Department of Medical Physics and Biomedical Engineering UCL London UK; ^5^ MRC Unit for Lifelong Health and Ageing at UCL London UK; ^6^ Leonard Wolfson Experimental Neurology Centre, Queen Square Institute of Neurology UCL London UK; ^7^ Neuroradiological Academic Unit, Department of Brain Repair and Rehabilitation UCL Queen Square Institute of Neurology London UK

**Keywords:** Alzheimer's disease, amyloid, biomarker, cognitively normal, cortical thickness, MRC National Survey of Health and Development, neurodegeneration, white matter hyperintensities

## Abstract

**Introduction:**

Cortical thickness has been proposed as a biomarker of Alzheimer's disease (AD)– related neurodegeneration, but the nature of its relationship with amyloid beta (Aβ) deposition and white matter hyperintensity volume (WMHV) in cognitively normal adults is unclear.

**Methods:**

We investigated the influences of Aβ status (negative/positive) and WMHV on cortical thickness in 408 cognitively normal adults aged 69.2 to 71.9 years who underwent ^18^F‐Florbetapir positron emission tomography (PET) and structural magnetic resonance imaging (MRI). Two previously defined Alzheimer's disease (AD) cortical signature regions and the major cortical lobes were selected as regions of interest (ROIs) for cortical thickness.

**Results:**

Higher WMHV, but not Aβ status, predicted lower cortical thickness across all participants, in all ROIs. Conversely, when Aβ‐positive participants were considered alone, higher WMHV predicted higher cortical thickness in a temporal AD‐signature region.

**Discussion:**

WMHV may differentially influence cortical thickness depending on the presence or absence of Aβ, potentially reflecting different pathological mechanisms.

## 1 INTRODUCTION

Cortical thickness has been proposed as a biomarker of neurodegeneration across the pathophysiological continuum of Alzheimer's disease (AD).^1^, ^2^, ^3^ It is vital to understand the pathological factors that may influence such biomarkers, particularly in asymptomatic individuals, who represent an increasingly important target population for potential disease‐modifying therapies^4^ and where objective biomarkers are likely to play key roles in the interpretation of such therapeutic trials in the future.^5^

Although the relationship between amyloid beta (Aβ) deposition^6^, ^7^, ^8^, ^9^, ^10^, ^11^, ^12^, ^13^, ^14^, ^15^, ^16^, ^17^, ^18^—one of the earliest hypothesized changes in the AD‐continuum^19^—and cortical thickness, as well as the relationship between white matter hyperintensity volume (WMHV)^20^, ^21^, ^22^, ^23^, ^24^—largely considered to be a surrogate marker of cerebral small vessel vasculopathy^25^—and cortical thickness, have been investigated in cognitively normal adults in isolation, there has been limited research looking at the relative influences of Aβ deposition and WMHV on cortical thickness concurrently.

We report a cross‐sectional analysis of 408 cognitively normal individuals (ages 69.2 to 71.9 years) all born in mainland Britain in the same week of 1946 who underwent clinical assessment, ^18^F‐Florbetapir positron emission tomography (PET) and volumetric structural magnetic resonance imaging (MRI). The aim of this analysis was to investigate the hypothesis that Aβ deposition and WMHV predict lower cortical thickness and to investigate potential interactions in their effects.

## 2 METHODS

### 2.1 Participants

Data were analyzed from individuals who participated in a neuroscience sub‐study (Insight 46) of the MRC National Survey of Health and Development (NSHD), a birth cohort study originally comprising 5362 individuals born in mainland Britain in 1 week of 1946.^26^ A total of 502 NSHD members were recruited to Insight 46 and underwent detailed clinical and neuropsychological assessment, MRI, and ^18^F‐florbetapir PET imaging.^27^, ^28^ Ethical approval was granted by the National Research Ethics Service Committee London (reference 14/LO/1173) and participants provided written informed consent.

Participants were assessed for a history of cognitive impairment, and major neurological or psychiatric illness. If a participant reported memory or cognitive difficulties they perceived as more than others the same age, or if they felt they would seek medical attention regarding cognitive difficulties, they were defined as having subjective memory complaints. A collateral cognitive history was acquired using the an eight‐item informant interview validated to differentiate aging and dementia (AD8) questionnaire^29^ and informant cognitive concern was defined as an AD8 score ≥2. Participants were defined as having mild cognitive impairment (MCI) if there was evidence of subjective cognitive concerns from the participant or the informant AND objective evidence of an amnestic (logical memory delayed recall score ≥1.5 standard deviations [SD] below the mean) or non‐amnestic cognitive deficit (digit‐symbol substitution score ≥1.5 standard deviations below the mean), AND there was no evidence of dementia. Logical memory delayed recall and digit‐symbol substitution were selected for this purpose, as they were both normally distributed.

Cognitive testing in Insight 46^27^, ^28^ included: the Mini‐Mental State Examination (MMSE), the digit‐symbol substitution test from the Wechsler Adult Intelligence Scale‐Revised, logical memory delayed recall from the Wechsler Memory Scale‐Revised, matrix reasoning from the Wechsler Abbreviated Scale of Intelligence, and the 12‐item Face‐Name test. Childhood cognitive function and educational attainment data were also available to characterize the sample. Childhood cognitive function was measured at age 8 (or age 11 or 15, if this was missing) as the sum of scores of four tests of verbal and non‐verbal ability standardized into a z‐score.^30^ Educational attainment was dichotomized into those with advanced (eg, "A level") or higher (eg, university) qualifications, versus those below this level.^31^ *APOE* genotype (ε4 carrier or non‐carrier), diabetes (based on a hemoglobin A1c (HbA1c) >6.5% at 65 years old, or prescription of diabetic medication at current assessment), hypertension (based on prescription of anti‐hypertensive agent or a blood pressure >140/90 mm Hg at current assessment), hypercholesterolemia (based on prescription of a cholesterol‐lowering agent) and smoking (smoked or never smoked based on questionnaire data aged 68 years) were also binarized to characterize the sample.

Exclusions from the analysis were the following: failure to complete the PET/MRI scan (n = 31); PET acquisition failure (n = 8); WMHV segmentation quality control failure (n = 4); cortical thickness imaging quality control failure (n = 3); and participants with evidence of dementia, MCI, or major neurological (including clinical history or radiological evidence of a cortical stroke) or psychiatric disorder (n = 48).

### 2.2 Florbetapir‐PET

Concurrent acquisition of PET and MRI was performed on the same Siemens Biograph mMR 3 Tesla PET/MRI scanner. Aβ burden was assessed during a 10‐minute period ≈50 minutes after injection of ≈370 MBq of ^18^F‐florbetapir. PET data were processed using an automated in‐house processing pipeline including pseudo‐CT (computed tomography) attenuation correction.^32^ A global standard uptake value ratio (SUVR) was calculated from a cortical gray matter composite (composed of the lateral and medial frontal, anterior and posterior cingulate, lateral parietal, and lateral temporal regions) using an eroded subcortical white matter reference region. Positive or negative Aβ status was determined using a Gaussian mixture model applied to SUVR values, taking the 99th percentile of the Aβ‐negative Gaussian as the cut‐point (0.6104).^28^

### 2.3 Structural MRI

MRI sequences pertaining to this analysis included volumetric T1‐weighted magnetization prepared rapid gradient echo (MPRAGE) images (voxel size 1.1 × 1.1 × 1.1 mm^3^ isotropic; inversion time/repetition time (TI/TR) = 870/2000 ms, total time = 5 minutes 6 seconds); volumetric T2‐weighted sampling perfection with application optimized contrasts using different flip angle evolution (SPACE) images (voxel size 1.1 × 1.1 × 1.1 mm^3^ isotropic; echo time (TE)/TR = 409/3200 ms, total time = 4 minutes 43 seconds); and volumetric fluid‐attenuated inversion recovery (FLAIR) inversion recovery sampling perfection with application optimized contrasts using different flip angle evolution (IR‐SPACE) images (voxel size 1.1 × 1.1 × 1.1 mm^3^ isotropic; TE/TI/TR = 402/1800/5000 ms, total time = 6 minutes 27 seconds). All MRI data were pre‐processed for gradwarp and image inhomogeneity.^27^

Cortical thickness estimation was performed using FreeSurfer version 6.0.^33^, ^34^ Two modifications to the standard automated pipeline were performed: a locally generated manually edited brain mask was used to improve skull stripping accuracy; and both T1‐weighted and T2‐weighted images were used as inputs to improve segmentation accuracy (https://surfer.nmr.mgh.harvard.edu/fswiki/recon-all#UsingT2orFLAIRdatatoimprovepialsurfaces).

**FIGURE 1:**
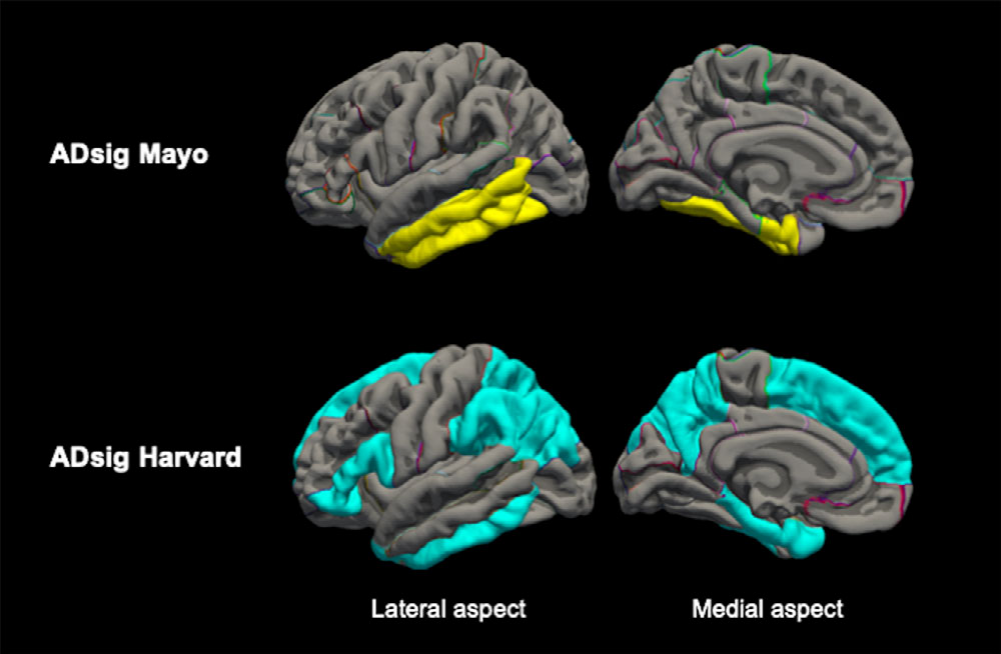
AD cortical signature composites used in the analysis. ADsig Mayo (top row), surface area–weighted mean of entorhinal cortex, fusiform, inferior, and middle temporal regions; ADsig Harvard (bottom row), surface area–weighted mean of entorhinal cortex, parahippocampus, inferior parietal lobe, pars opercularis, pars orbitalis, pars triangularis, inferior temporal, temporal pole, precuneus, supramarginal gyrus, superior parietal, and superior frontal regions; AD, Alzheimer's disease

Cortical thickness regions of interest (ROIs) included surface area–weighted averages of two previously proposed composite signatures of AD cortical thinning (Figure 1): one based on work by Jack and colleagues^1^ comprising select temporal cortex regions (entorhinal, fusiform, inferior, and middle temporal—“ADsig Mayo”), and one based on work by Dickerson and colleagues,^2^, ^3^, ^35^, ^36^ which includes select frontal, temporal and parietal regions (entorhinal cortex, parahippocampus, inferior parietal lobe, pars opercularis, pars orbitalis, pars triangularis, inferior temporal lobe, temporal pole, precuneus, supramarginal gyrus, superior parietal lobe, and superior frontal lobe—“ADsig Harvard”). In addition, we generated ROIs for surface area–weighted averages of the four major cortical lobes (frontal, temporal, parietal, and occipital—https://surfer.nmr.mgh.harvard.edu/fswiki/corticalParcellation) to assess broad trends across the whole cortex (see Table 1).

**HIGHLIGHTS**

- Cortical thickness is a biomarker of neurodegeneration in Alzheimer's disease
- In 408 healthy 70‐year‐olds amyloid beta (Aβ) positivity did not predict cortical thickness
- Overall, higher white matter hyperintensity volume predicted lower cortical thickness
- There was evidence this relationship differed in the context of Aβ deposition

**RESEARCH IN CONTEXT**

Systematic review: The authors searched PubMed for articles assessing the relationship between cortical thickness and both amyloid beta (Aβ) deposition, and white matter hyperintensity volume (WMHV). Although, cortical thickness is a widely applied biomarker of neurodegeneration across the pathophysiological continuum of Alzheimer's disease (AD), there has been limited research looking at the relative influences of Aβ deposition and WMHV on cortical thickness concurrently.

Interpretation: We found that WMHV is a stronger predictor of cortical thickness than Aβ deposition in cognitively normal 70‐year‐olds. Furthermore, we found that WMHV may be differentially associated with cortical thickness according to the extent of Aβ deposition. This has implications for the use of cortical thickness as a biomarker of neurodegeneration in the preclinical phase of AD and suggests that WMHV may reflect different pathological mechanisms in the presence of Aβ accumulation.

**TABLE 1:** Regions from the FreeSurfer Desikan atlas contributing to surface area–weighted averages of major cortical lobe cortical thickness data

Lobe	Regions from the FreeSurfer Desikan atlas—https://surfer.nmr.mgh.harvard.edu/fswiki/corticalParcellation
Frontal	Superior frontal, rostral middle frontal, caudal middle frontal, pars opercularis, pars triangularis, pars orbitalis, lateral orbitofrontal, medial lateral orbitofrontal, precentral, paracentral, frontal pole, rostral anterior cingulate, and caudal anterior cingulate
Temporal	Superior temporal, middle temporal, and inferior temporal, banks of the superior temporal sulcus, fusiform, transverse temporal, entorhinal, temporal pole, and parahippocampal
Parietal	Superior parietal, inferior parietal, supramarginal, postcentral, precuneus, posterior cingulate, and isthmus cingulate
Occipital	Lateral occipital, lingual, cuneus, and pericalcarine

Bayesian Model Selection (BaMoS),^25^ a validated automated segmentation tool that uses jointly volumetric T1‐weighted and FLAIR MRI data, was used to generate an estimate of WMHV. This generates a global value that includes subcortical gray matter but excludes infratentorial regions; more detail of how BaMoS was applied to Insight 46 data has been described in detail elsewhere.^37^

### 2.4 Statistical approach

Wilcoxon rank‐sum tests, or where the outcome was normally distributed, two‐sample *t* tests were used to compare continuous clinical and cognitive characteristics between Aβ‐positive and Aβ‐negative groups. Logistic regression models were used to compare categorical variables between Aβ‐positive and Aβ‐negative groups. Spearman correlation coefficients were used to assess unadjusted relationships between WMHV and continuous variables. Wilcoxon rank‐sum tests were used to test associations between WMHV and categorical variables.

Linear regression models were used to test the hypothesis that Aβ status, and global WMHV independently predicted cortical thickness in each cortical ROI (dependent variable), adjusting for sex and age at time of scan. A similar analysis using global SUVR as a continuous measure of Aβ load to predict cortical thickness was also performed (covariates = WMHV, sex, and age at time of scan). In addition, the interaction term between Aβ status and global WMHV was added to each model to test the hypothesis that the two interact in terms of their effect on cortical thickness. In the cortical ROIs where there was evidence of a significant interaction, linear regression was then used to test the hypothesis that global WMHV was associated with cortical thickness in each ROI in the Aβ‐negative and Aβ‐positive populations separately, adjusting for sex and age at time of scan. Q‐Q plots and residual‐versus‐fitted plots were generated to test the assumptions that the outcome variables and their residuals were normally distributed with constant variance. Robust standard errors were used to account for non‐constant variance. All analyses were performed using Stata version 14.

## 3 RESULTS

### 3.1 Sample characterization

Seventy‐four of 408 (18.1%) of cognitively normal individuals were Aβ positive. *APOE* genotype strongly predicted Aβ status, with ε4 carriers being five times more likely to be Aβ positive. There were no statistically significant differences between Aβ‐positive and Aβ‐negative individuals in the following: age at time of scan, WMHV, sex, childhood cognitive ability, educational attainment, logical memory, digit‐symbol substitution scores, 12‐item Face‐Name test score, diabetes, hypercholesterolemia, or smoking. Matrix reasoning and MMSE scores were marginally lower in the Aβ‐positive group compared to the Aβ‐negative group, as reported previously (see Table 2).^28^

**TABLE 2:** Sample characterization

	Aβ‐negative (n = 334)	Aβ‐positive (n = 74)	Aβ‐negative vs Aβ‐positive	Unadjusted association with WMHV(n = 408)
Age, years, median (IQR)	70.7 (1.2)	70.7 (1.1)	*P* = 0.66^a^	Rho = 0.12; *P* = 0.013*,^b^
Male sex, n (%)	166 (49.7%)	40 (54.1%)	OR 0.84; *P* = 0.5^c^	*P* = 0.17^a^
Earliest childhood cognitive ability, z‐score	0.41 (0.74)	0.44 (0.75)	*P* = 0.74^d^	Rho = −0.05; *P* = 0.27^b^
Advanced level of education, n (%)	181 (54.2%)	37 (50%)	OR 0.85; *P* = 0.51^c^	*P* = 0.86^a^
MMSE, median (IQR), maximum score = 30	30 (1)	29 (1)	*P* = 0.063^a^	Rho = −0.03; *P* = 0.52^b^
Logical memory score, mean (SD), maximum score = 25	11.7 (3.6)	11.3 (3.7)	*P* = 0.33^d^	Rho = −0.05; *P* = 0.35^b^
Digit‐symbol substitution score, mean (SD), maximum score = 93	48.8 (10.1)	46.9 (9.7)	*P* = 0.14^d^	Rho = −0.11; *P* = 0.03*,^b^
Matrix reasoning, median (IQR), maximum score = 32	26 (4)	25 (4)	*P* = 0.037* ^,^ a	Rho = −0.04; *P* = 0.47^b^
12‐Item Face‐Name test, median (IQR), maximum score = 96	66 (28)	68 (27)	*P* = 0.29^a^	Rho = 0.05; *P* = 0.34^b^
*APOE* ε4 carrier, n (%) (missing data: n = 2)	76 (22.9%)	45 (60.8%)	OR 5.22; *P* < 0.0001^c^	*P* = 0.49^a^
Hypertension, n (%) (missing data: n = 1)	217 (64.9%)	42 (57.5%)	OR 0.73; *P* = 0.23^c^	*P* = 0.004* ^,^ a
Hypercholesterolemia, n (%)	116 (34.7%)	28 (37.8%)	OR 1.14; *P* = 0.61^c^	*P* = 0.22^a^
Diabetes, n (%) (missing data: n = 4)	35 (10.6%)	9 (12.1%)	OR 1.17; *P* = 0.7^c^	*P* = 0.14^a^
Ever‐smoked, n (%)	216 (64.7%)	46 (62.1%)	OR 0.89; *P* = 0.68^c^	*P* = 0.32^a^
WMHV, milliliters, median (IQR)	2.85 (4.84)	3.30 (4.97)	*P* = 0.48^a^	‐

Wilcoxon rank‐sum tests, or two‐sample *t* tests if the outcome was normally distributed, were used to compare continuous clinical and cognitive characteristics between Aβ‐positive and Aβ‐negative groups. Logistic regression models were used to compare categorical variables between Aβ‐positive and Aβ‐negative groups. Spearman correlation coefficients were used to assess unadjusted relationships between WMHV and continuous variables. Wilcoxon rank‐sum tests were used to test associations between WMHV and categorical variables. IQR, interquartile range; MMSE, Mini‐Mental State Examination; OR, odds ratio; WMHV, white matter hyperintensity volume (milliliters).

a Wilcoxon rank‐sum test.

b Spearman rank correlation.

c Logistic regression.

d *t* test.

^*^ = *P* < 0.05.

In unadjusted analyses, there was no evidence of an association at the 5% level between WMHV and sex, childhood cognitive ability, educational attainment, MMSE, logical memory delayed recall, matrix reasoning, 12‐item Face‐Name test score, *APOE* genotype, diabetes, hypercholesterolemia, or smoking (Table 2). There was some evidence of a positive association between greater age at time of scan and WMHV (*P* = 0.013), a negative association between digit‐symbol substitution score and WMHV (*P* = 0.03), and that individuals with a history of hypertension had higher WMHV (*P* = 0.004).

### 3.2 Cortical thickness and Aβ deposition

There was no evidence that Aβ status or SUVR predicted cortical thickness in any cortical ROI investigated following adjustment for age at time of scan, sex, and WMHV (Table 3). To explore whether adjustment for WMHV had removed a statistically significant association between Aβ cortical thickness, the same analysis without adjustment for WMHV was performed. However, this made no material difference to the results obtained (data not shown).

**TABLE 3:** Association between Aβ‐deposition (binary: positive/negative status, and continuous: SUVR classification) and cortical thickness in Insight 46

ROIs	Aβ‐negative mean cortical thickness (SD), mm	Aβ‐positive mean cortical thickness (SD), mm	Mean difference (95% CI), mm	*P*‐value	β‐Coefficient between SUVR and cortical thickness	*P*‐value
Frontal	2.76 (0.09)	2.75 (0.09)	−0.01 (−0.03, 0.01)	0.32	−0.10 (−0.24, 0.05)	0.18
Temporal	2.86 (0.09)	2.87 (0.09)	0.00 (−0.02, 0.03)	0.71	−0.03 (−0.15, 0.09)	0.64
Parietal	2.47 (0.08)	2.48 (0.09)	0.01 (−0.01, 0.03)	0.32	−0.02 (−0.15, 0.11)	0.82
Occipital	2.19 (0.09)	2.20 (0.1)	0.01 (−0.02, 0.03)	0.65	−0.07 (−0.22, 0.08)	0.37
ADsig Mayo	2.89 (0.09)	2.89 (0.09)	0.01 (−0.02, 0.03)	0.59	−0.05 (−0.18, 0.08)	0.48
ADsig Harvard	2.68 (0.07)	2.69 (0.08)	0.00 (−0.02, 0.02)	0.83	−0.05 (−0.17, 0.08)	0.44

Mean difference calculated following linear regression analysis with robust standard error. β‐Coefficients were adjusted for age at scan, sex, and WMHV.

Aβ, amyloid β; ADsig Mayo, surface area–weighted mean cortical thickness in entorhinal cortex, fusiform, inferior and middle temporal regions; ADsig Harvard, surface area–weighted mean cortical thickness in entorhinal cortex, parahippocampus, inferior parietal lobe, pars opercularis, pars orbitalis, pars triangularis, inferior temporal, temporal pole, precuneus, supramarginal gyrus, superior parietal, and superior frontal regions; ROIs, regions of interest; SUVR, standard uptake value ratio; WMHV, white matter hyperintensity volume.

### 3.3 Cortical thickness and WMHV

Across all participants, there was evidence that WMHV predicted lower cortical thickness in all ROIs investigated independent of Aβ deposition, age at time of scan, and sex. In addition, there was evidence of a *positive* interaction between Aβ status and WMHV in terms of their effects on cortical thickness in the temporal lobe and ADsig Mayo ROIs, and to a lesser extent the frontal lobe (Table 4).

**TABLE 4:** Association between WMHV and cortical thickness in Insight 46

ROIs	β‐Coefficient between WMHV and cortical thickness†	*P*‐value	Interaction term between Aβ status and WMHV†	*P*‐value
Frontal	−3.55 (−5.07, −2.03)	<0.001*	3.65 (0.66, 6.65)	0.017*
Temporal	−3.3 (−4.81, −1.79)	<0.001*	5.77 (2.8, 8.72)	<0.001*
Parietal	−1.24 (−2.41, −0.06)	0.039*	−0.29 (−3.56, 2.97)	0.86
Occipital	−1.89 (−3.39, −0.39	0.013*	1.91 (−2.63, 6.47)	0.41
ADsig Mayo	−1.99 (−3.5, −0.47)	0.01*	6.07 (3.11, 9.04)	<0.001*
ADsig Harvard	−1.51 (−2.59, −0.44)	0.006*	1.17 (−1.56, 3.89)	0.4

β‐Coefficients were adjusted for age at scan, sex, and Aβ status.

Aβ, amyloid β; ADsig Mayo, surface area–weighted mean cortical thickness in entorhinal cortex, fusiform, inferior, and middle temporal regions; ADsig Harvard, surface area–weighted mean cortical thickness in entorhinal cortex, parahippocampus, inferior parietal lobe, pars opercularis, pars orbitalis, pars triangularis, inferior temporal, temporal pole, precuneus, supramarginal gyrus, superior parietal, and superior frontal regions; ROIs, regions of interest; SUVR, standard uptake value ratio; WMHV, white matter hyperintensity volume.

^*^= *P* < 0.05.

^†^= mm/ml × 10^−3^.

In ROIs where there was evidence of an interaction between Aβ status and WMHV, we also stratified participants into Aβ‐negative and Aβ‐positive groups to test if WMHV differentially influenced cortical thickness in the two groups. In Aβ‐negative individuals alone there was strong evidence of an association between increasing global WMHV and lower cortical thickness in all ROIs investigated after adjusting for age at scanning and sex. However, in the Aβ‐positive group, a negative association between WMHV and cortical thickness was not clearly evident, and there was evidence that higher global WMHV was associated with increased cortical thickness in the ADsig Mayo ROI (*P* = 0.027) (Table 5).

**TABLE 5:** Association between WMHV and cortical thickness in Insight 46 following stratification based on Aβ status

ROIs	β‐Coefficient between WMHV and cortical thickness Aβ‐negative, n = 334†	*P*‐value	β‐Coefficient between WMHV and cortical thickness Aβ‐positive, n = 74†	*P*‐value
Frontal	−4.21 (−5.89, −2.53)	<0.001*	−0.56 (−3.2, 2.07)	0.67
Temporal	−4.35 (−5.9, −2.8)	<0.001*	1.3 (−1.2, 0.39)	0.29
ADsig Mayo	−3.13 (−4.66, −1.6)	<0.001*	2.9 (0.33, 5.47)	0.027*

β‐Coefficients were adjusted for age at scan and sex.

Aβ, amyloid β; ADsig Mayo, surface area–weighted mean cortical thickness in entorhinal cortex, fusiform, inferior, and middle temporal regions; ADsig Harvard, surface area–weighted mean cortical thickness in entorhinal cortex, parahippocampus, inferior parietal lobe, pars opercularis, pars orbitalis, pars triangularis, inferior temporal, temporal pole, precuneus, supramarginal gyrus, superior parietal, and superior frontal regions; ROIs, regions of interest; SUVR, standard uptake value ratio; WMHV, white matter hyperintensity volume.

^*^= *P* < 0.05.

^†^ = mm/mL × 10^−3^.

In a post hoc analysis we also explored whether the patterns observed were related to *APOE* genotype by adding *APOE* ε4 carrier status as a covariate, as well an interaction term between *APOE* ε4 carrier status and WMHV, to the statistical models and this made no material difference to the results obtained. Given the possibility that WMHV may reflect different pathological mechanisms in different contexts, we also explored whether hypertension predicted WMHV differentially in Aβ‐negative and Aβ‐positive groups using Wilcoxon rank‐sum tests. Aβ‐Negative individuals with a history of hypertension had higher WMHV (*P* = 0.0017), whereas there was no evidence of an association in Aβ‐positive participants (*P* = 0.84).

## 4 DISCUSSION

In this study of 408 age‐matched cognitively individuals, we found no evidence, even at a trend level, for cross‐sectional associations between Aβ deposition and cortical thickness in any of the ROIs investigated. Although some studies have shown evidence of associations between Aβ deposition and cortical thickness in cognitively normal individuals,^7^, ^11^, ^12^, ^13^, ^35^ several studies have also reported null findings.^14^, ^15^, ^16^, ^17^ Because AD is associated with cortical thinning, the lack of association in this cohort may reflect the age of the participants investigated. These participants were just at the beginning of the eighth decade, and at this age the majority of Aβ‐positive individuals are likely to be in a relatively early stage of the pathophysiological continuum of AD,^38^, ^39^, ^40^ and so potentially many years before the onset of AD‐related neurodegeneration. Many previous studies have utilized data sets with older age ranges, which increases the probability of including participants closer to symptom onset and consequently with more significant AD‐related neurodegeneration. A further important consideration when evaluating inconsistencies between studies in the literature is the large array of methodological factors that influence PET‐derived quantitative estimates of Aβ deposition.^41^ However, it is worth noting that the rate of Aβ positivity in this cohort was consistent with meta‐analyses of Aβ‐PET imaging studies of cognitively normal individuals,^38^ which increases confidence in the reliability of the methodology utilized in this study.

Although we found no relationship between Aβ deposition and cortical thickness, higher WMHV strongly predicted lower cortical thickness across all participants. This is consistent with previous studies, which have provided evidence for cross‐sectional associations between increasing WMHV and gray matter atrophy^20^, ^21^, ^22^, ^23^, ^24^; this study confirms and extends these findings, allowing for associations with Aβ deposition to be explored.

We found evidence for an interaction between Aβ status and WMHV in terms of their effects on cortical thickness. When Aβ‐positive participants were considered alone, there was some evidence that higher WMHV predicted *higher* cortical thickness in the ADsig Mayo ROI. Although the statistical effect in Aβ‐positive participants was relatively weak (*P* = 0.027), the interaction effect between Aβ status and WMHV across all participants was strong (*P* < 0.001 in temporal ROIs). Although paradoxical increases in cortical gray matter structural metrics with Aβ deposition have been reported in cognitively normal individuals,^8^, ^9^, ^10^, ^18^ to our knowledge, a positive association between WMHV and cortical thickness has not been reported previously in individuals with asymptomatic Aβ deposition. However, a similar interaction in the setting of clinically established AD consistent with our findings, also localized to the temporal lobe, has been reported.^42^

The mechanism(s) leading to positive associations between WMHV and cortical thickness are unclear. Elevated WMHV may reflect pathological processes other than conventional cerebrovascular disease in the context of AD.^43^, ^44^ In a large cohort of non‐demented older adults, Graff‐Radford et al. found white matter hyperintensities to be correlated with Aβ load, with evidence implicating cerebral amyloid angiopathy rather than small vessel vasculopathy.^45^ It is notable that in our study hypertension was strongly related to WMHV in the Aβ‐negative population, consistent with this being due to small vessel vasculopathy, whereas there was no evidence of an association in the Aβ‐positive sample, noting differences in sample size between Aβ‐negative and Aβ‐positive groups. Another possibility for this unexpected relationship is neuroinflammation. Recent work has demonstrated positive associations between PET radiotracer uptake of markers of microglial activation and gray matter volumes in the setting of MCI.^46^ It is also possible that this is reflective of a selection effect, whereby those with a thicker cortex are more likely to be recruited to an intensive neuroimaging study and be defined as cognitively normal in the context of a higher WMHV load and Aβ positivity. Longitudinal imaging and neuropsychology data will be important to further explore the consequence of this observed effect, whereas ultimately large‐scale post‐mortem data will be vital to investigate further the pathological heterogeneity underlying white matter hyperintensities in the context of Aβ deposition.

This study has a number of strengths and weaknesses. Because all subjects were of near identical age—separated only by the ≈2 to 3 years required to complete scanning—the effects of age on any relationships are limited. We do not have a measure of neurofibrillary tangle deposition such as tau PET, which is more closely linked to neurodegeneration and cognitive decline than Aβ^47^, ^48^; recent work has shown that tau PET tracer uptake is strongly correlated with lower cortical thickness and may mediate relationships between Aβ and cortical atrophy.^49^ However, the study by Graff‐Radford and colleagues demonstrated no relationship between increased tau and white matter hyperintensity burden,^45^ suggesting that it is unlikely to play a role in the interactions between Aβ and WMHV in terms of their effects on cortical thickness in our data set. We considered Aβ and WMHV on a global scale, and future work looking at regional differences in pathological biomarkers and cortical thickness will be of interest, as will work looking at relevant white matter pathways using diffusion MRI.^50^ Although the broader NSHD is highly representative of individuals born in mainland Britain in 1946,^26^ intensive neuroimaging studies such as Insight 46 are at risk of introducing an element of recruitment bias; we have previously shown that Insight 46 participants are on average slightly more educated, as well as more likely to being defined as having a non‐manual occupation.^51^ However, compared to many other intensive data‐rich neuroimaging studies, which rely on convenience sampling methodology,^52^ this is likely to remain a fairly representative samples, noting that all NSHD participants are white, which is reflective of the British population in 1946, but not the richer ethnic diversity present in the modern United Kingdom.

In conclusion, we found that WMHV is a stronger predictor of cortical thickness than Aβ deposition in normal 70‐year‐olds. This, and the demonstration that WMHV may be differentially associated with cortical thickness according to Aβ status, has implications for the use of cortical thickness as a biomarker of AD‐related neurodegeneration in the preclinical phase and suggests that WMHV may reflect different pathological mechanisms in the presence of Aβ accumulation.
